# A Case of Tegafur-Uracil-Induced Interstitial Lung Disease Presenting as Hypersensitivity Pneumonitis

**DOI:** 10.7759/cureus.103741

**Published:** 2026-02-16

**Authors:** Manato Taguchi, Takumi Kiwamoto, Kai Kawashima, Kazuhumi Yoshida, Nobuyuki Hizawa

**Affiliations:** 1 Department of Pulmonary Medicine, University of Tsukuba, Tsukuba, JPN

**Keywords:** di-ild, dlst, drug-induced interstitial lung disease, drug-induced lymphocyte stimulation test, hypersensitivity pneumonia, lung cancer, rectal cancer, tegafur-uracil

## Abstract

This report describes the case of a 77-year-old man undergoing chemotherapy for synchronous lung cancer and rectal cancer. After receiving tegafur-uracil (UFT) plus leucovorin in combination with bevacizumab for rectal cancer, he developed exertional dyspnea. Chest computed tomography revealed diffuse bilateral centrilobular nodules and ground-glass opacities, suggesting a hypersensitivity pneumonia (HP) pattern of drug-induced interstitial lung disease (DI-ILD), and he was therefore admitted for further evaluation and treatment. Bronchoalveolar lavage demonstrated an increased lymphocyte fraction with a decreased CD4/CD8 ratio, and a drug-induced lymphocyte stimulation test was positive only for UFT. Based on the clinical course and these findings, the patient was diagnosed with UFT-induced interstitial lung disease. His respiratory condition improved rapidly following corticosteroid therapy. DI-ILD associated with UFT is rare, and to the best of our knowledge, no previous reports have described an HP pattern on imaging. We therefore report this case with a review of the relevant literature.

## Introduction

Drug-induced interstitial lung disease (DI-ILD) is a collective term for noninfectious respiratory disorders that develop during drug administration and encompasses a wide spectrum of clinical manifestations, most commonly interstitial pneumonia [1,2]. The proposed mechanisms include direct toxicity of the drug or its metabolites, immunologically mediated hypersensitivity reactions, and dysregulated cytokine production [1,2]. Although imaging findings are generally nonspecific, high-resolution computed tomography (HRCT) typically demonstrates diffuse ground-glass opacities, reticular abnormalities, and interlobular septal thickening. Based on radiologic patterns, DI-ILD is commonly classified into nonspecific interstitial pneumonia, organizing pneumonia, diffuse alveolar damage, hypersensitivity pneumonitis (HP), and simple pulmonary eosinophilia [1,2].

In the treatment of lung cancer, platinum-based agents, taxanes, epidermal growth factor receptor tyrosine kinase inhibitors (EGFR-TKIs), and immune checkpoint inhibitors (ICIs) are frequently used, all of which are known to cause DI-ILD at a certain incidence [3,4]. In contrast, tegafur-uracil (UFT), an oral fluoropyrimidine anticancer agent, is widely used as adjuvant chemotherapy and for the treatment of gastrointestinal malignancies, but reports of UFT-induced interstitial lung disease (ILD) are relatively rare [5-8]. To date, no cases of UFT-induced ILD presenting with an HP pattern on imaging have been reported in patients with lung cancer.

Here, we report a case of drug-induced pneumonitis with an HP pattern on imaging that developed during UFT therapy for synchronous lung cancer and rectal cancer, and compare it with previously reported cases in the literature.

## Case presentation

A 77-year-old man was admitted with exertional dyspnea while undergoing treatment for synchronous lung adenocarcinoma and rectal cancer. His medical history included hypertension, hyperlipidemia, and diabetes mellitus. He smoked 25 cigarettes per day for 56 years until he was 76 years of age. Although no definite history of antigen exposure was identified, the patient reported a possible history of asbestos exposure during his employment in the construction industry.

One year before admission, abdominal computed tomography (CT) performed for the evaluation of left epididymitis revealed abdominal lymphadenopathy. Subsequent positron emission tomography (PET)-CT was conducted to investigate the underlying cause, which identified tumor lesions in the right upper lobe of the lung and in the rectum. Biopsy specimens from both lesions revealed adenocarcinoma; however, immunohistochemical staining showed distinct profiles. The lung lesion was positive for cytokeratin 7 (CK7) and thyroid transcription factor-1 (TTF-1), and negative for CK20, Napsin A, surfactant protein A (SP-A), CDX2, and p40, whereas the rectal lesion was negative for CK7 and positive for CK20 and CDX2. Based on these findings, the patient was diagnosed with synchronous lung adenocarcinoma (EGFR mutation-negative, ALK immunohistochemistry-negative, and PD-L1 tumor proportion score >90%) and rectal cancer. 

**Figure 1:**
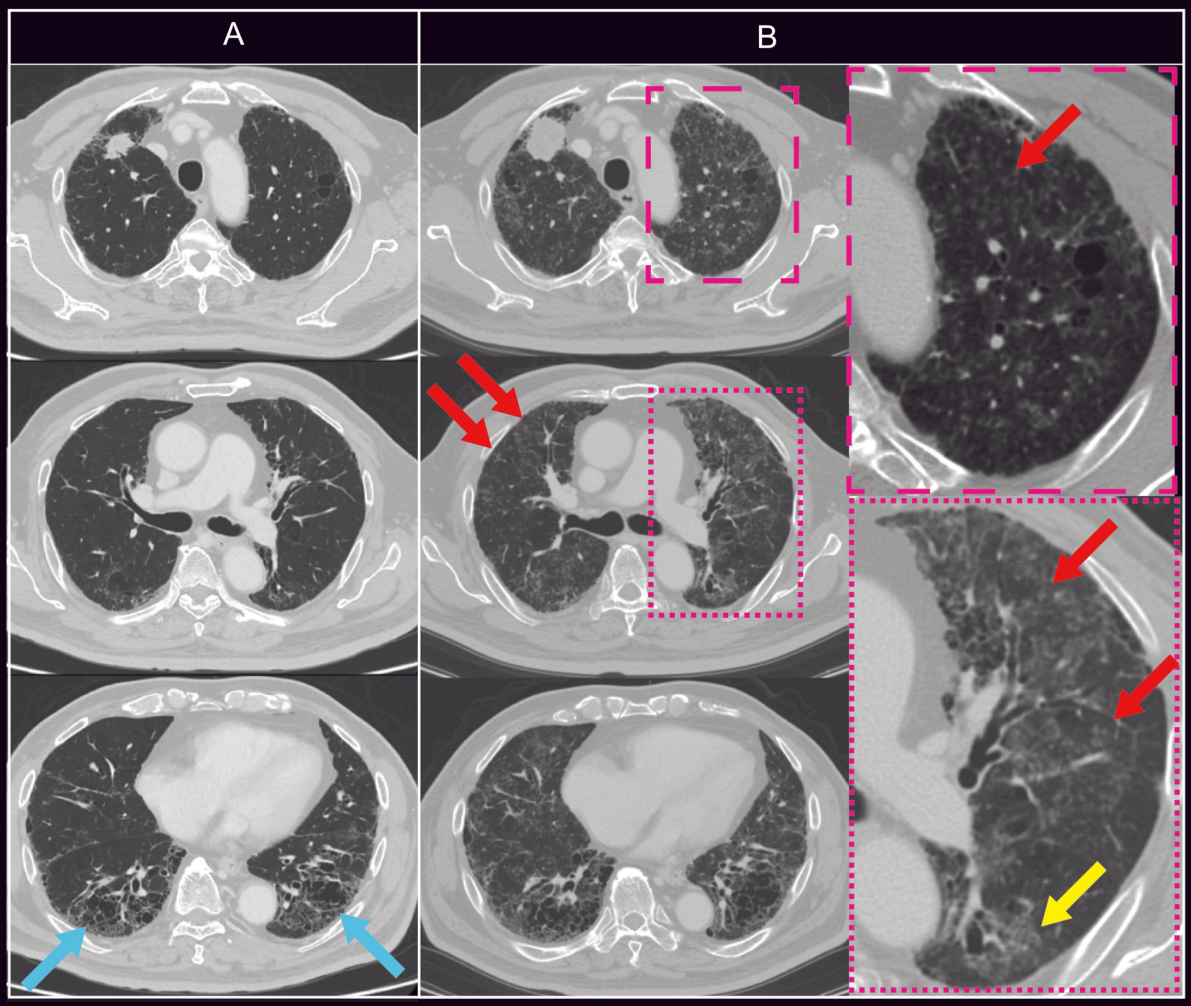
Chest CT images obtained before initiation of UFT/LV plus bevacizumab (A) and three months after treatment initiation, at the onset of dyspnea (B) Before treatment initiation, fibrosis and honeycombing were observed in both lower lung fields (blue arrows). After the onset of dyspnea, newly developed centrilobular nodules (red arrows) and ground-glass opacities (yellow arrows) were noted in both lungs. UFT: tegafur–uracil; LV: leucovorin

During stoma creation to relieve stenosis caused by the rectal tumor, perigastric lymph nodes were sampled, and histopathological examination revealed lymph node metastasis originating from the lung cancer. Accordingly, the lung cancer was staged as cT3N0M1b, stage IV. Although systemic chemotherapy was planned for stage IV lung adenocarcinoma, ICI therapy was withheld for the following reasons: (i) pre-existing interstitial lung abnormalities with honeycombing and fibrosis in both lung bases before treatment initiation (Figure 1A); (ii) an elevated Krebs von den Lungen-6 (KL-6) level of 885 U/mL despite negative autoantibodies and surfactant protein-D (SP-D) levels; and (iii) reduced vital capacity (VC) and diffusing capacity for carbon monoxide (DL_co_) (Table 1). Collectively, these findings suggested the coexistence of interstitial pneumonia. Eight months before admission, the patient received four cycles of carboplatin plus paclitaxel chemotherapy for lung cancer, achieving a partial response. However, progression of the rectal cancer was observed. Therefore, considering the patient’s advanced age and a performance status equivalent to PS 1, treatment with UFT plus leucovorin (LV) in combination with bevacizumab was initiated. 

**Table 1:** Pulmonary function test VC: vital capacity; FEV_1.0_: forced expiratory volume in one second; FVC: Forced vital capacity; TLC: total lung capacity; RV: residual volume; DL_CO_: diffusing capacity for carbon monoxide; DLCO/VA: diffusing capacity of the lung for carbon monoxide per unit alveolar volume

Parameter (Unit)	One year before admission	At admission	Reference values
VC (L)	2.49	2.05	3.31
%VC (%)	75.3	61.9	>80
FEV_1.0_ (L)	1.91	1.98	2.53
%FEV_1.0_ (%)	75.2	78.3	
FEV_1.0_/FVC (%)	79.3	92.5	>70
TLC (L)	4.24	3.09	5.15
%TLC (%)	82.5	60	
RV (L)	1.75	1.04	1.74
%RV (%)	101.7	59.8	
DL_CO_	11.28	5.29	14.04
%DL_CO _(%)	78.9	37.7	>80
DL_CO_/VA	3.4	2.18	4.11
%DL_CO_/VA (%)	82.1	53	>80

Approximately three months after the initiation of treatment, the patient developed exertional dyspnea that did not improve with antibiotic therapy. Chest CT revealed enlargement of the primary lung tumor and metastatic lesions, as well as diffuse bilateral centrilobular nodules and ground-glass opacities, raising suspicion of DI-ILD (Figure 1B). However, despite discontinuation of UFT/LV plus bevacizumab, his respiratory status showed little improvement, and he was therefore admitted for further evaluation and treatment of suspected DI-ILD. 

On admission, the patient’s body temperature was 36.8°C, blood pressure was 116/57 mmHg, and heart rate was 105 beats per minute, indicating tachycardia. Oxygen saturation was 93% on room air, and he reported exertional dyspnea corresponding to a modified Medical Research Council (mMRC) grade 3. On physical examination, fine crackles were auscultated over both lung fields, and no lower extremity edema was observed. Laboratory examinations revealed mild elevations in C-reactive protein and lactate dehydrogenase, along with increased levels of KL-6 and SP-D (Table 2). Arterial blood gas analysis showed a pH of 7.411, partial pressure of arterial oxygen (PaO₂) of 66.3 Torr, partial pressure of carbon dioxide (PaCO₂) of 39.8 Torr, and bicarbonate (HCO₃⁻) of 24.8 mEq/L, with an increased alveolar-arterial oxygen gradient (A-aDO₂) of 33.7. Pulmonary function testing demonstrated marked deterioration in VC, total lung capacity (TLC), residual volume (RV), and DL_co_ compared with values obtained one year prior to admission (Table 1). Bronchoalveolar lavage revealed an increased total cell count of 3.9 × 10⁵ cells/mL, with a differential of 27.6% macrophages, 66.5% lymphocytes, 2.9% neutrophils, and 2.9% eosinophils, along with a markedly decreased CD4/CD8 ratio of 0.1. However, transbronchial lung biopsy (TBLB) revealed only mild fibrosis and lymphocytic infiltration and was insufficient to establish a histopathological diagnosis of a specific ILD subtype. No organisms suggestive of infection were identified. These findings were consistent with DI-ILD. A drug-induced lymphocyte stimulation test (DLST) performed to identify the causative agent was positive for UFT (319%) and negative for LV (133%).

**Table 2:** Laboratory findings on admission WBC: white blood cell; Neu: neutrophil; Lym: lymphocyte; Mono: monocyte; Eos: eosinophil; Baso: basophil; RBC: red blood cell; Hb: hemoglobin; Hct: hematocrit; Plt: platelet; Alb: albumin; AST: aspartate aminotransferase; ALT: alanine aminotransferase; LDH: lactate dehydrogenase; ALP: alkaline phosphatase; γ-GTP: gamma‑glutamyl transpeptidase; T-Bil: total bilirubin; Na: sodium; K: potassium; Cl: chloride; BUN: blood urea nitrogen; Cre: creatinine; eGFR: estimated glomerular filtration rate; CRP: C-reactive protein; Ca: calcium; HbA1c (NGSP): Hemoglobin A1c (NGSP); NT-proBNP: N-terminal pro–B-type natriuretic peptide ; KL-6: Krebs von den Lungen-6; SP-D: Surfactant protein-D; Ig: Immunoglobulin; RF: Rheumatoid factor; Anti-CCP antibody: Anti–cyclic citrullinated peptide antibody; Anti-ARS antibody: Anti–aminoacyl–tRNA synthetase antibody; PR3-ANCA: Proteinase 3–antineutrophil cytoplasmic antibody; MPO-ANCA: Myeloperoxidase–antineutrophil cytoplasmic antibody. Reference ranges are shown in the rightmost column for comparison.

Parameter (Unit)	Patient Value	Reference Range
WBC (/μL)	9800	4500-9000
Neu (%)	75.5	49-62
Lym (%)	17.6	25-45
Eos (%)	6.1	1-5
Baso (%)	0.8	0-1
RBC (×10⁴/µL)	385	427-570
Hb (g/dL)	11.5	14-18
Hct (%)	35.6	40-52
Plt (×10⁴/µL)	33.7	15-35
Alb (g/dL)	3.3	3.8-5.3
AST (U/L)	16	8-38
ALT (U/L)	5	4-44
LDH (U/L)	274	106-211
ALP (U/L)	190	104-338
γ-GTP (U/L)	16	12-63
T-Bil (mg/dL)	0.4	0.3-1.2
Na (mEq/L)	139	135-147
Cl (mEq/L)	104	98-108
K (mEq/L)	4.4	3.6-5
BUN (mg/dL)	15.2	8-20
Cre (mg/dL)	1.01	0.61-1.04
eGFR (mL/min)	55.2	60-100
CRP (mg/dL)	1.46	0-0.2
Ca (mg/dL)	9.2	8.6-10.1
Blood Sugar (mg/dL)	163	70-199
HbA1c(NGSP) (%)	7.3	4.6-6.2
NTproBNP (pg/ml)	27.56	0-125
D-dimer (μg/ml)	3.5	0-1
KL-6 (U/ml)	2127	0-499
SP-D (ng/ml)	265	0-109
IgG (mg/dL)	1361	870-1700
IgA (mg/dL)	426	110-410
IgM (mg/dL)	114	33-190
IgE (U/ml)	27	0-170
RF (U/ml)	15	0-15
Anti-CCP antibody	0.5	0-4.4
Antinuclear antibody (titer)	40	0-39
Homogeneous pattern	Negative	
Speckled pattern	Positive	
Centromere pattern	Negative	
Nucleolar pattern	Negative	
Peripheral pattern	Negative	
Granular pattern	Negative	
Nuclear membrane pattern	(-)	
Anti-SS-A antibody	(-)	
Anti-SS-B antibody	(-)	
Anti-Scl-70 antibody	(-)	
Anti-Jo-1 antibody	(-)	
Anti-ARS antibody	(-)	
PR3-ANCA	<1.0	0-3.4
MPO-ANCA	<1.0	0-3.4
β-D-glucan (pg/mL)	<6.0	0-10.9

**Figure 2:**
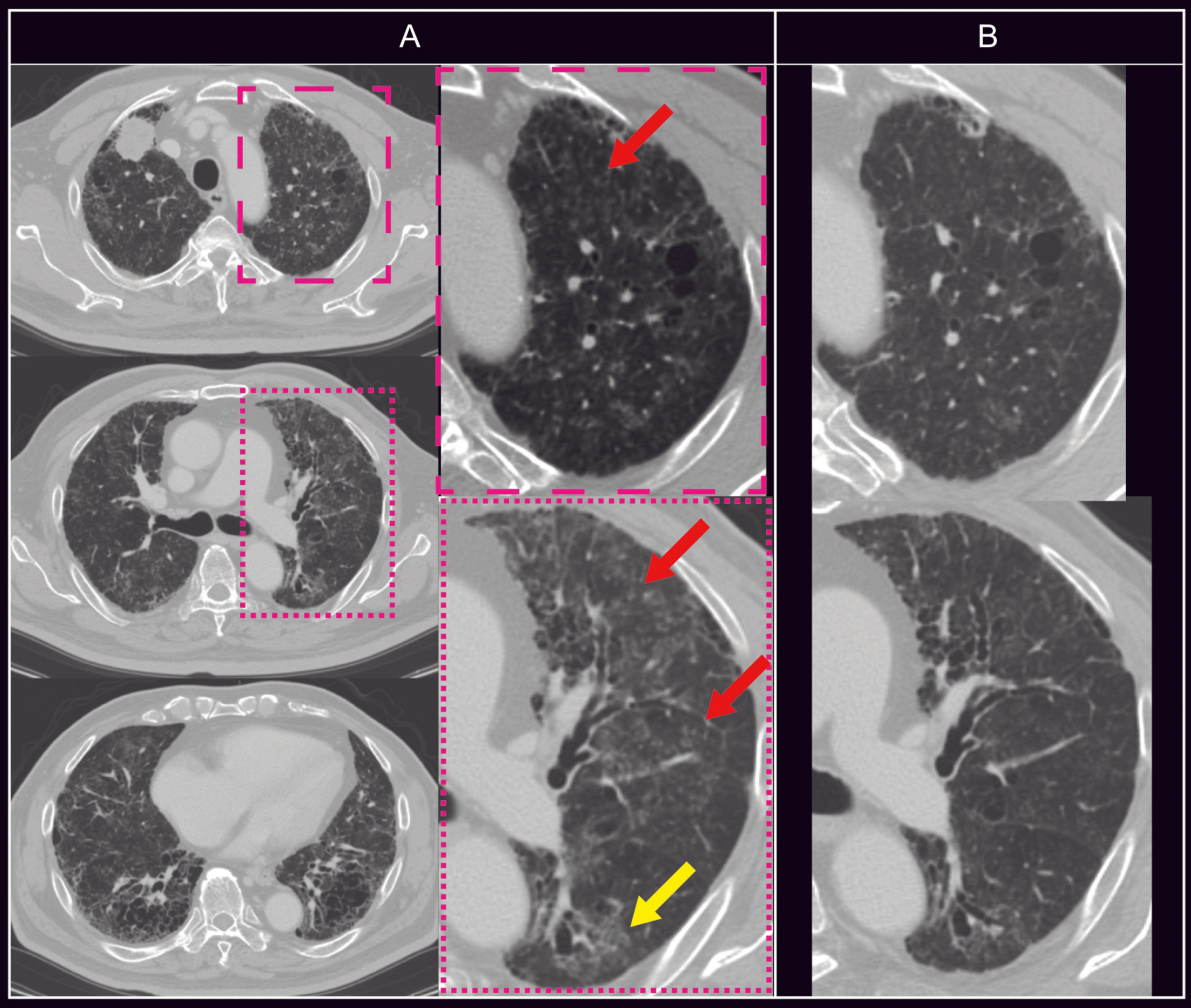
Chest CT obtained three months after UFT/LV plus bevacizumab treatment initiation at the onset of dyspnea (A), and two weeks after oral corticosteroid therapy (B) Following two weeks of oral corticosteroid therapy, the centrilobular nodules (A, red arrows) and ground-glass opacities (A, yellow arrows) showed marked improvement. UFT: tegafur–uracil; LV: leucovorin; OCS: oral corticosteroid therapy

Based on these findings, the patient was diagnosed with UFT-induced ILD, and oral prednisolone was initiated at a dose of 40 mg/day (0.5 mg/kg/day). His subjective symptoms improved within several days after the start of treatment, and follow-up CT performed two weeks later demonstrated marked improvement of the diffuse bilateral pulmonary opacities (Figure 2). In contrast, both lung cancer and rectal cancer showed disease progression.

Although progression of the lung cancer was considered the major determinant of prognosis, a decision was made to pursue best supportive care for the following reasons: (i) despite a trend toward improvement, disease control of DI-ILD was insufficient to allow tapering of systemic corticosteroids to approximately 10 mg/day; and (ii) the combination regimen of carboplatin plus paclitaxel, which is relatively commonly used in patients with non-small cell lung cancer complicated by underlying ILD, had already proven ineffective, while TS-1 was not considered an appropriate option because of its pharmacological overlap with UFT, conferring a high risk of exacerbating DI-ILD with limited expected therapeutic benefit. He was discharged on day 30 after the initiation of corticosteroid therapy, following the introduction of home oxygen therapy and gradual tapering of the prednisolone dose to 20 mg/day. Thereafter, no recurrence of DI-ILD was observed; however, the patient died four months after discharge due to progression of the underlying malignancies. 

## Discussion

This case represents DI-ILD that developed after the initiation of UFT therapy and was diagnosed based on an integrated assessment of the clinical course, imaging findings, bronchoalveolar lavage (BAL) results, and DLST. To the best of our knowledge, there have been no previous reports of UFT-associated DI-ILD presenting with HP pattern on imaging, as observed in the present case.

The diagnosis of DI-ILD cannot be established by a single test and requires a comprehensive evaluation of multiple factors, including: (i) a temporal relationship with drug administration, (ii) exclusion of alternative diagnoses such as infection, heart failure, and lymphangitic carcinomatosis, (iii) consistency between imaging and BAL findings, (iv) clinical improvement after discontinuation of the suspected drug; and (v) recurrence upon re-exposure to the drug [1,2]. In this case, respiratory symptoms refractory to antibiotic therapy developed approximately three months after the initiation of UFT. Chest CT demonstrated diffuse centrilobular nodules and ground-glass opacities consistent with an HP pattern. BAL revealed lymphocytosis with a decreased CD4/CD8 ratio. Furthermore, the patient showed rapid clinical improvement following drug withdrawal and corticosteroid therapy. Collectively, these findings strongly supported a diagnosis of DI-ILD.

Among anticancer agents used for the treatment of lung cancer, taxanes, EGFR-TKIs, and ICIs are known to cause DI-ILD relatively frequently, with reported incidences in non-small cell lung cancer of 4.6%, 0-5.7%, and 0-10.6%, respectively [4]. In contrast, the reported incidence of UFT-induced DI-ILD ranges from less than 0.1% [6], and only a small number of cases have been described in case reports (Table 3) [7-11]. Accordingly, UFT is generally considered to have low pulmonary toxicity; however, fatal cases due to severe lung injury have been reported, warranting careful attention [7]. In the present case, DI-ILD developed three months after the initiation of UFT, which was consistent with the timing reported in previous cases. However, (i) in patients with ILD, the presence of severe dyspnea corresponding to an mMRC grade of 3, as observed in this case, is known to be associated with impaired diffusing capacity and a poor prognosis [12,13], necessitating prioritization of steroid therapy; and (ii) both lung cancer and rectal cancer had shown a tendency toward progression during treatment. For these reasons, re-administration of UFT, even at a reduced dose, was not pursued. On the other hand, the presence of pre-existing ILD and the use of combination chemotherapy may increase the risk of DI-ILD [1]. In this context, it is conceivable that administering combination chemotherapy, including UFT for the treatment of rectal cancer in a patient with pre-existing ILD, may have contributed to an increased risk of DI-ILD onset.

**Table 3:** Summary of UFT-induced interstitial lung disease case reports ILD: interstitial lung disease; DLST: drug-induced lymphocyte stimulation test; DAD: diffuse alveolar damage; HP: hypersensitivity pneumonitis; EP: eosinophilic pneumonia; UFT: tegafur-uracil; Ad: adenocarcinoma; Sq: squamous cell carcinoma

Author, Year	Age (years)	Sex	Primary Cancer	Onset (months)	ILD pattern	DLST	Treatment	Clinical Outcome
Numata, et al., 1995 [9]	54	F	Breast Cancer	2	ILD (not otherwise specified)	Positive (430%)	Steroid	Survived
Yamashiro, et al., 1995 [10]	83	M	Pharynx Cancer	2	ILD (not otherwise specified)	Positive (318%)	Steroid	Survived
Ootani, et al., 2003 [11]	60	M	Lung Cancer (Ad)	9	ILD (not otherwise specified)	Positive	Discontinue UFT	Survived
Nakashima and Shibata, 2006 [7]	72	M	Lung Cancer (Sq)	0.5	DAD	Positive (432%)	Steroid	Died
Nagahiro, et al., 2011 [8]	68	F	Lung Cancer (Ad)	0.5	EP	Positive (288%)	Steroid	Survived
Present Case	77	M	Lung Cancer (Ad) and Rectum Cancer	3	HP	Positive (319%)	Steroid	Survived

Radiologic patterns of DI-ILD are classified into DAD, OP, NSIP, and HP, among others [1,2]. Cases presenting with an HP pattern on imaging are generally considered to be more responsive to corticosteroid therapy [2]. In addition, typical BAL findings in DI-ILD include lymphocytic alveolitis with CD8-positive T-cell predominance [1]. In this case, both the imaging findings and BAL results were consistent with DI-ILD, suggesting a high likelihood of steroid responsiveness. Early initiation of corticosteroid therapy was therefore undertaken, resulting in prompt clinical improvement.

DLST is widely used in Japan as an adjunctive diagnostic tool for DI-ILD; however, its diagnostic utility is limited [2]. The median positivity rate of DLST in patients with drug-induced pneumonitis has been reported to be 66.9%, but test performance varies depending on the characteristics of the suspected drug, and both sensitivity and specificity differ substantially among agents [2]. For example, antituberculosis drugs are reported to have high specificity but low sensitivity, indicating that a negative result does not exclude the causative drug [14]. Furthermore, fluoropyrimidine-based anticancer agents containing 5-fluorouracil, such as TS-1 and UFT, may activate the salvage pathway, a DNA recycling mechanism. Consequently, there is concern that cells exposed to these agents may yield false-positive DLST results, even when the examined cells are not actively proliferating [15,16]. Therefore, DLST alone is not recommended for the definitive diagnosis of DI-ILD, and in the present case, the possibility that the UFT result was biased toward a false-positive reaction should be considered. Meanwhile, Kawabata et al. also reported that, among 20 healthy volunteers, six showed DLST positivity to TS-1 [16]. Although the cutoff value for DLST positivity was defined as ≥180%, consistent with the present case, five of these six cases demonstrated values below 250%, corresponding to false-positive to mildly positive ranges. Furthermore, only one of the 20 subjects exhibited a markedly elevated DLST value exceeding 300%. Therefore, when DLST results for other suspected agents are negative, and the DLST value for UFT is markedly elevated, as observed in the present case, such a result, when interpreted in conjunction with the clinical course and other findings, may reasonably support the identification of UFT as the suspected causative drug.

Based on these backgrounds, the DLST result in this case was considered supportive of UFT as the causative agent because: (i) only UFT showed a markedly elevated DLST value of 319%, which far exceeded both the standard DLST positivity threshold of 180% and the cutoff for false-positivity of 200%, whereas all other tested agents yielded negative results; and (ii) the clinical course, imaging findings, and BAL results were all consistent with DI-ILD. Unfortunately, in this case, the synchronous malignancies progressed during treatment for DI-ILD, and there was no indication for re-administration of UFT, LV, or bevacizumab; therefore, complete exclusion of other potential causative agents was difficult. As no effective subsequent anticancer therapy was available, the patient was transitioned to best supportive care. Nevertheless, prompt therapeutic intervention following the diagnosis of DI-ILD, together with a comprehensive evaluation integrating the treatment history, radiological findings, blood tests such as DLST, BAL fluid analysis, and histopathological findings to identify the suspected causative drug, is expected to provide clinically meaningful benefits by facilitating the continuation of lung cancer treatment.

## Conclusions

This case demonstrates that DI-ILD caused by UFT can present with a HP pattern on imaging. Although the diagnosis of DI-ILD requires a comprehensive assessment based on clinical information, the diagnostic utility of DLST should be carefully evaluated for each agent, with particular attention to the potential for false-positive or false-negative results, before it is considered supportive evidence for causality. When unexplained interstitial pneumonia develops during anticancer therapy, clinicians should always consider the possibility of DI-ILD, even with agents considered to have a low incidence of pulmonary toxicity, and should pursue early diagnosis and prompt therapeutic intervention.
